# A case of hemobilia caused by a pseudoaneurysm resulting in obstructive cholangitis in a patient who underwent plastic stent placement for pancreatic cancer (with video)

**DOI:** 10.1002/deo2.70130

**Published:** 2025-04-29

**Authors:** Yusuke Seyama, Akinori Sugaya, Ken Koyama, Hiroyuki Honda, Hajime Ishibashi, Masaharu Yamaoka, Mitsuharu Ozeki, Mitsuhide Goto, Masahiro Araki, Kenji Amagai

**Affiliations:** ^1^ Department of Gastroenterology Ibaraki Prefectural Central Hospital Ibaraki Japan; ^2^ Department of Medical Oncology Ibaraki Prefectural Central Hospital Ibaraki Japan; ^3^ Department of Diagnostic and Interventional Radiology Ibaraki Prefectural Central Hospital Ibaraki Japan

**Keywords:** biliary plastic stent, hemobilia, obstructive cholangitis, pseudoaneurysm, transcatheter arterial embolization

## Abstract

Hemobilia, or hemorrhage within the biliary system, is an uncommon cause of stent obstruction associated with bile duct strictures that are rarely observed with plastic stents. Reports of a pseudoaneurysm as a cause of hemobilia after plastic stent placement are also rare. We present a rare case of hemobilia caused by a pseudoaneurysm that resulted in obstructive cholangitis in a patient who underwent plastic stent placement for bile duct invasion caused by pancreatic cancer. A 78‐year‐old man with a history of stroke who was using antiplatelet therapy presented with obstructive jaundice caused by locally advanced pancreatic cancer and underwent endoscopic plastic stent placement in the bile duct followed by concurrent chemoradiotherapy. Second‐line chemotherapy was administered as the disease progressed; however, he experienced obstructive cholangitis and was admitted to our hospital. Hemobilia was identified as the underlying cause; notably, it was not evident during the initial evaluation and was diagnosed during endoscopic stent replacement. Emergency angiography revealed a pseudoaneurysm of the posterior superior pancreaticoduodenal artery, which was successfully treated using coil embolization. Cholangitis and hemobilia resolved, and the patient was discharged without bleeding recurrence.

## INTRODUCTION

Endoscopic stenting is a well‐established treatment for jaundice in patients with malignant biliary obstruction. Although stent patency is often limited^1^ by factors such as biliary sludge and biofilm formation, hemobilia is a rare cause of stent obstruction, especially with plastic stents. Additionally, few reports of pseudoaneurysm formation and subsequent hemobilia after plastic stent placement have been published. We report a unique case of obstructive cholangitis caused by hemobilia attributable to a pseudoaneurysm in a patient who underwent plastic stent placement because of pancreatic cancer. Notably, hemobilia was not evident until the time of endoscopic stent replacement, which complicated its diagnosis.

## CASE REPORT

**FIGURE 1:**
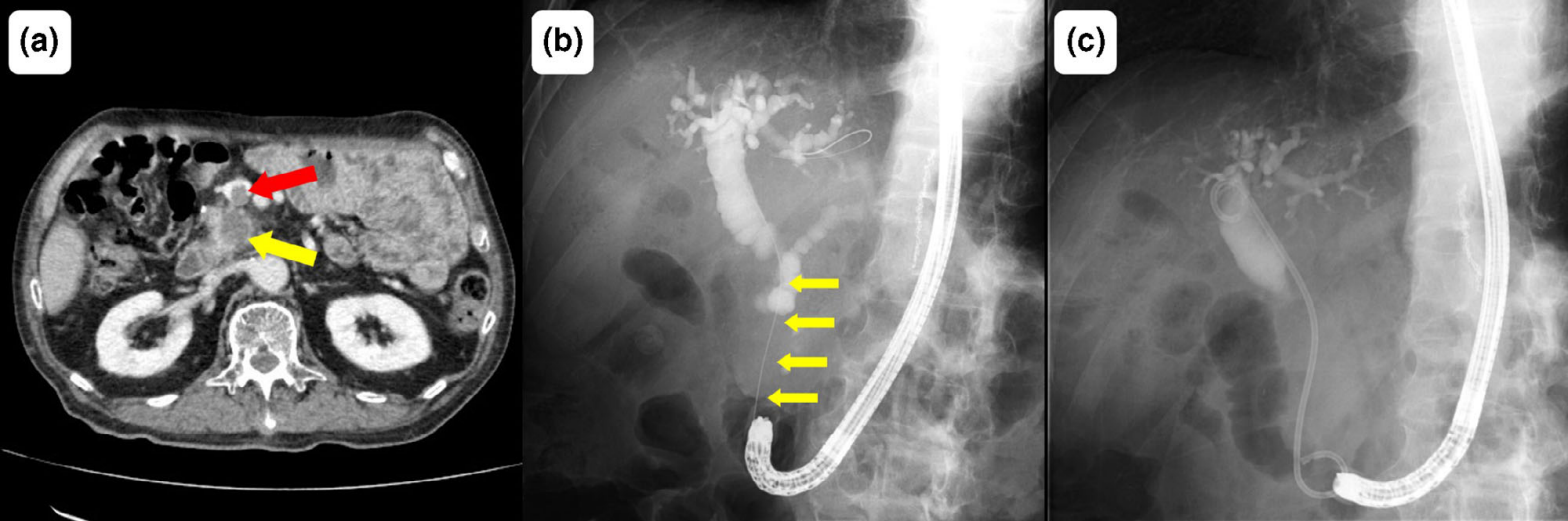
Imaging findings prior to hemobilia. (a) Image obtained during contrast‐enhanced computed tomography showing pancreatic cancer (yellow arrow) and portal vein tumor thrombosis (red arrow). (b) Initial radiographic image obtained during endoscopic retrograde cholangiopancreatography revealing stenosis (approximately 6 cm) in the distal bile duct (yellow arrow). (c) Radiographic image obtained during endoscopic retrograde cholangiopancreatography performed at the onset of cholangitis. A bilateral pigtail plastic stent is placed in the bile duct.

The patient was a 78‐year‐old man with comorbid hyperuricemia, diabetes mellitus, and a history of stroke who was using clopidogrel for secondary prevention. His surgical history included pyloric gastrectomy, cholecystectomy, splenectomy, and Billroth II reconstruction for gastric cancer 19 years prior without evidence of recurrence. The patient was referred to our hospital with clinical features of obstructive jaundice and portal vein tumor thrombosis caused by pancreatic head cancer (Figure 1a). Endoscopic retrograde cholangiopancreatography (ERCP) revealed stenosis (6 cm) in the distal bile duct (Figure 1b), and cytopathological examination of the site confirmed adenocarcinoma consistent with pancreatic cancer invasion. A plastic stent was placed to improve jaundice. The tumor was considered unresectable because of superior mesenteric artery invasion and the presence of portal vein tumor thrombosis. Radiation therapy (50 Gy in 25 fractions) and S‐1 (tegafur/gimeracil/oteracil) chemotherapy were initiated. After the development of liver metastasis, second‐line treatment comprising gemcitabine plus nab‐paclitaxel was administered. Two episodes of cholangitis occurred during treatment; however, both resolved with plastic stent replacement and antibiotics (Figure 1c). Notably, bleeding was not observed during stent replacement.

**FIGURE 2:**
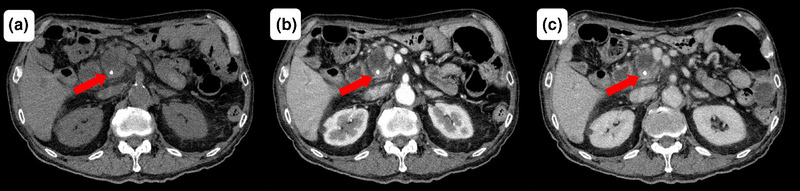
Contrast‐enhanced computed tomography findings before endoscopic retrograde cholangiopancreatography. Contrast extravasation is not observed before the replacement of the plastic stent (red arrow). (a) Plain computed tomography image. (b) Delayed arterial phase. (c) Portal phase.

**FIGURE 3:**
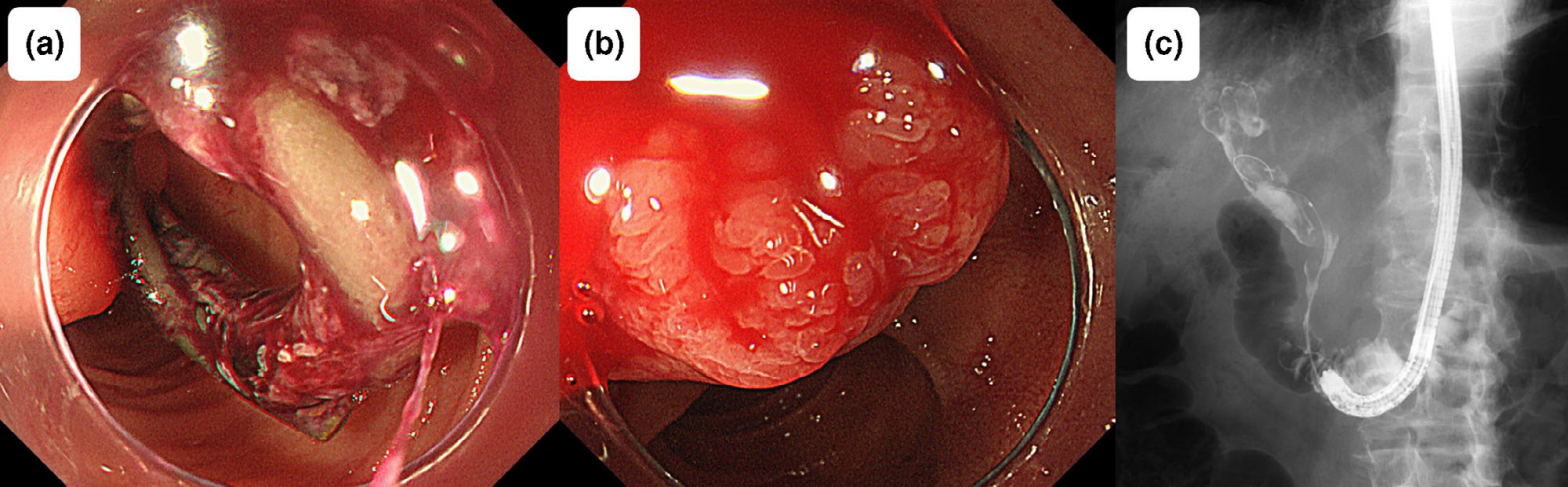
Endoscopic retrograde cholangiopancreatography findings of hemobilia. (a) Endoscopic image of the plastic stent covered with blood clots and without visible bile outflow. (b) Endoscopic image of the papilla of Vater after stent removal showing pulsatile bleeding from the papilla. (c) Radiographic image obtained during endoscopic retrograde cholangiopancreatography revealing multiple filling defects within the bile ducts.

**FIGURE 4:**
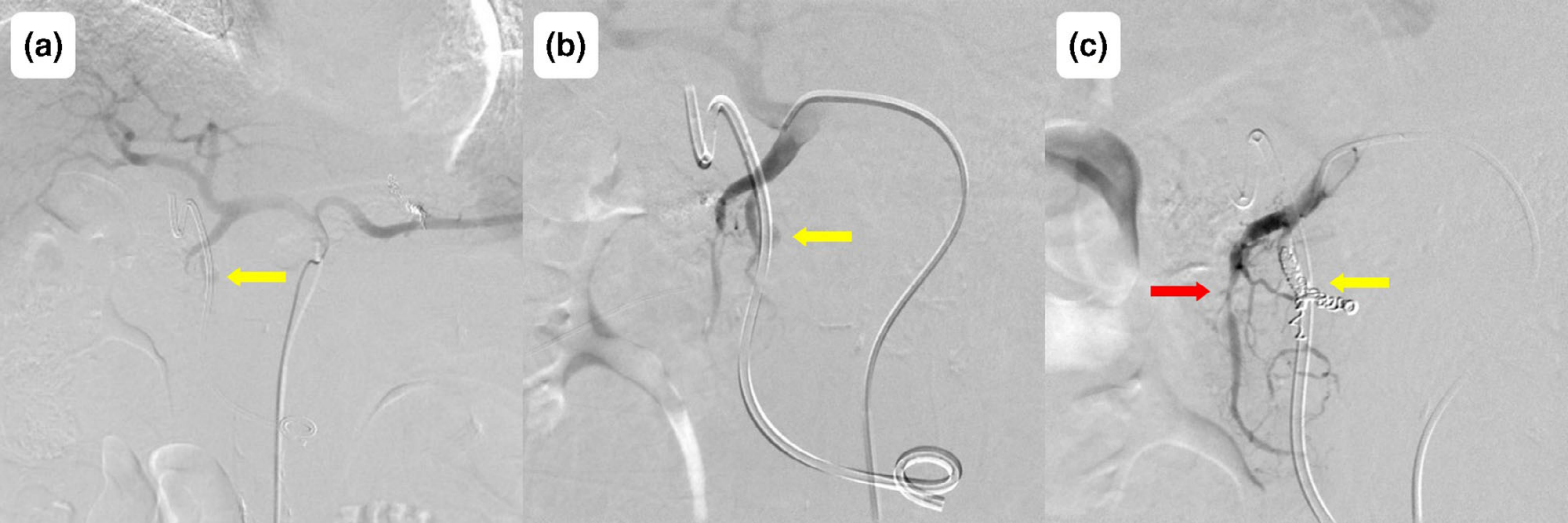
Angiographic findings of hemobilia. (a) Angiographic image of the celiac artery showing a pseudoaneurysm in a branch of the gastroduodenal artery (yellow arrow). (b) Angiographic image of the gastroduodenal artery demonstrating a pseudoaneurysm of the posterior superior pancreaticoduodenal artery (yellow arrow). (c) Angiographic image of the gastroduodenal artery after coil embolization showing successful resolution of the pseudoaneurysm (yellow arrow). Irregular stenosis is also observed in the anterior superior pancreaticoduodenal artery (red arrow).

Fifty‐one days after the most recent replacement of the plastic stent in the bile duct, the patient presented to our emergency department with chills and upper abdominal pain. On admission, his vital signs were as follows: body temperature, 38.1°C; blood pressure, 76/58 mmHg; and pulse rate, 138 beats per minute. Laboratory findings revealed leukocytosis (9700/µL), mild anemia (hemoglobin level of 9.1 g/dL, which was not significantly changed from 8.9 g/dL observed 1 week previously), an elevated C‐reactive protein level (which is an inflammatory marker; 1.85 mg/dL), and hepatobiliary enzyme abnormalities (aspartate aminotransferase, 192 U/L; alanine aminotransferase, 46 U/L; alkaline phosphatase, 728 U/L; γ‐glutamyl transpeptidase, 429 U/L; total bilirubin, 1.3 mg/dL). Contrast‐enhanced computed tomography (CT) revealed intrahepatic bile duct dilatation without plastic stent displacement or extravasation (Figure 2a–c). Septic shock caused by cholangitis was diagnosed, and the patient was hospitalized. Antibiotics and vasopressors were initiated, and emergency ERCP (Video S1) was performed after blood pressure stabilization. ERCP revealed a blood clot on the plastic stent (Figure 3a), no bile outflow, and pulsatile bleeding from the papilla of Vater after stent removal (Figure 3b). Cholangiography revealed multiple intraductal filling defects (Figure 3c), thus confirming obstructive cholangitis secondary to hemobilia. After the new plastic stent was placed, contrast‐enhanced CT showed no evidence of extravasation into the bile duct (Figure S1a–c), prompting emergency angiography. Angiography revealed a pseudoaneurysm in the posterior superior pancreaticoduodenal artery as the bleeding source (Figure 4a,b). Coil embolization of the posterior superior pancreaticoduodenal artery was successful, and the bleeding stopped (Figure 4c) without anemia progression. Antibiotics and vasopressors were gradually discontinued, and the patient was discharged 20 days after admission without repeated stent occlusion.

## DISCUSSION

This rare case had two novel features. First, a pseudoaneurysm, which results in hemobilia, is rare in patients who undergo plastic stent placement. Second, symptoms of cholangitis preceded any findings suggestive of bleeding, and hemobilia was evident only during endoscopic stent replacement. Hemobilia has various etiologies, including iatrogenic causes, malignancy, chronic ductal obstruction, and intraductal infection.^2^ The distribution of these causes has shifted over time, with malignancies accounting for approximately 7% of all cases as of 2001.^2^ Among 4579 patients with pancreatic ductal adenocarcinoma, the incidence of nonpostoperative gastrointestinal bleeding was 1.6%, and hemobilia accounted for only 2.1% of these cases.^3^ Although self‐expandable metallic stents for malignant biliary strictures have been associated with hemobilia,^4^ its occurrence after plastic stent placement is rare. Additionally, hemobilia as a cause of stent occlusion is rare.

In our patient, hemobilia originated from a pseudoaneurysm in the posterior superior pancreaticoduodenal artery. The exact mechanism remains unclear; however, several factors associated with the formation of pseudoaneurysms in the bile ducts have been proposed. These factors include mechanical damage and arterial wall necrosis caused by physical compression attributable to self‐expandable metallic stent placement,^5^ angiogenesis and fragmentation of vascular mesothelial elastic fibers along with edema of subcutaneous blood vessels induced by radiation therapy,^6^ and inflammation extending to the arterial wall caused by chronic cholangitis.^7^ Pseudoaneurysm formation associated with plastic stents is rare; only a few cases involving the hepatic artery because of mechanical compression at the stent edge have been reported.^8^ In our case, the bleeding occurred at the central part rather than at the edge of the stent. However, because extravasation of contrast medium was not observed with contrast‐enhanced CT after ERCP, mechanical compression by the stent at the site of pseudoaneurysm formation was suggested. Additionally, angiography revealed irregular stenosis of the anterior superior pancreaticoduodenal artery, indicating vascular invasion by pancreatic cancer. Based on these findings, we considered that multiple factors—stent compression, radiation therapy, and cholangitis—damaged blood vessels that were already weakened by cancer invasion, resulting in hemobilia. Typical symptoms of hemobilia include jaundice, right upper quadrant abdominal pain, and gastrointestinal bleeding. However, these three events occur simultaneously in only 22%–35% of cases.^2^ In our case, fever and abdominal pain were the presenting symptoms; interestingly, anemia and CT evidence of contrast extravasation were not initially observed. Therefore, the initial diagnosis of hemobilia was difficult. Extravasation was not observed, even after stent replacement, likely because the plastic stent compressed the bleeding site. Hypotension on admission was attributed to cholangitis rather than anemia because vasopressor treatment was still required after red blood cell transfusion. To our knowledge, no previous report has documented hemobilia after plastic stent placement for pancreatic cancer preceded by cholangitis without overt bleeding symptoms.

Treatment of hemobilia includes endoscopic therapy, transcatheter arterial embolization, and surgery. Endoscopic hemostasis can be achieved via full‐coverage self‐expandable metallic stents or balloon tamponade^2^; however, in this case, hemobilia was apparent only during stent replacement. Because of the urgency of this case and the need for precise treatment, transcatheter arterial embolization was chosen as treatment. Despite its risks such as ischemic liver injury,^2^ transcatheter arterial embolization is highly effective, with a 100% technical success rate reported for iatrogenic hemobilia caused by percutaneous transhepatic biliary drainage.^9^

In summary, we encountered a rare case of obstructive cholangitis caused by hemobilia as a result of a pseudoaneurysm in a patient who underwent plastic stent placement for pancreatic cancer. Successful treatment was achieved with plastic stent replacement and transcatheter arterial embolization; however, hemobilia was not evident until the time of stent replacement. Although hemobilia caused by a pseudoaneurysm is rare, it should be considered a potential complication after plastic stent placement and a possible cause of stent obstruction.

## CONFLICT OF INTEREST STATEMENT

None.

## ETHICS STATEMENT

Approval of the research protocol by an Institutional Reviewer Board: This case report was approved by the ethics committee of Ibaraki Prefectural Central Hospital.

## PATIENT CONSENT STATEMENT

The patient provided consent for publication of this case report through an institutional opt‐out consent procedure.

## CLINICAL TRIAL REGISTRATION

N/A.

## Supporting information




**VIDEO S1** Video of the endoscopic retrograde cholangiopancreatography procedure demonstrating hemobilia.

**FIGURE S1** Contrast‐enhanced computed tomography (CT) findings immediately after endoscopic retrograde cholangiopancreatography (ERCP). Contrast extravasation is not observed after the replacement of the plastic stent (PS; red arrow). a) Plain CT image. b) Early arterial phase. c) Portal phase
