# Supplementary figures and images for: A case of hemobilia caused by a pseudoaneurysm resulting in obstructive cholangitis in a patient who underwent plastic stent placement for pancreatic cancer (with video)
Source: DEN Open. 2025 Apr 29;6(1):e70130. doi: 10.1002/deo2.70130 (PMC12038176; doi:10.1002/deo2.70130)

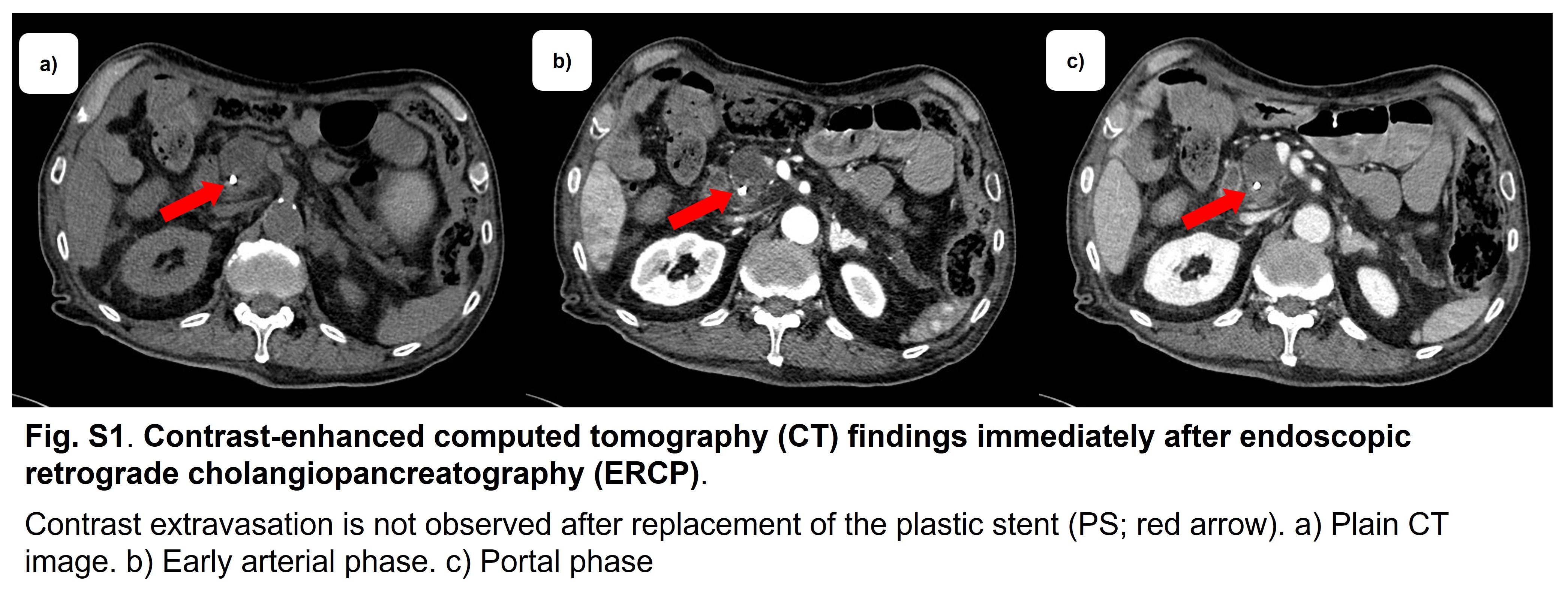

Supplement: Supplementary file 2 — FIGURE S1 Contrast‐enhanced computed tomography (CT) findings immediately after endoscopic retrograde cholangiopancreatography (ERCP). Contrast extravasation is not observed after the replacement of the plastic stent (PS; red arrow). a) Plain CT image. b) Early arterial phase. c) Portal phase [file DEO2-6-e70130-s001.jpg]
